# Quantitative assessment of photic phenomena in the presbyopia-correcting intraocular lens

**DOI:** 10.1371/journal.pone.0260406

**Published:** 2021-12-01

**Authors:** Yuki Ukai, Hajime Okemoto, Yusuke Seki, Yui Nakatsugawa, Akane Kawasaki, Teppei Shibata, Tsuyoshi Mito, Eri Kubo, Hiroshi Sasaki

**Affiliations:** Department of Ophthalmology, Kanazawa Medical University, Kahoku, Ishikawa, Japan; Saarland University, GERMANY

## Abstract

This was a prospective study to evaluate the feasibility of the photic phenomena test (PPT) for quantifying glare, halo, and starburst. We compared two presbyopia-correcting intraocular lenses (IOLs), the Symfony IOL and the PanOptix IOL, as well as the monofocal Clareon IOL in 111 IOL-implanted eyes of 111 patients who underwent the PPT 1 month postoperatively. The reproducibility of photic phenomena with the PPT was assessed in 39 multifocal IOL-implanted eyes of 20 patients and among the examiners. Patients with ocular diseases, except for refractive errors, were excluded. The mean values of the groups were evaluated. Bland–Altman plots were used to analyze statistical data (Easy R version 1.37; R Foundation for Statistical Computing, Vienna, Austria). The PPT reproducibility assessment revealed no fixed bias or regressive significance. Reproducibility was confirmed. The glare size did not differ significantly between the Symfony, PanOptix, and Clareon groups. The halo size was significantly larger in the Symfony group (p < 0.01) than in the PanOptix group. The halo intensity was significantly brighter in the PanOptix group (p < 0.01) than in the Symfony group. In contrast, no halos were perceived in the Clareon group. The starburst size or intensity did not differ significantly between the Symfony, PanOptix, and Clareon groups. We identified the photic phenomenon related to various IOLs.

## Introduction

The recent advent of intraocular lenses (IOLs), such as toric and presbyopia-correcting IOLs, has increased patients’ expectations regarding the postoperative quality of vision after undergoing modern cataract surgery [1]. Presbyopia-correcting IOLs are highly effective in providing an extended field of vision and improving the patients’ quality of life [2]. Conversely, photic phenomena, such as glare, halo, and starburst, have been reported in eyes implanted with presbyopia-correcting IOLs, resulting in patient dissatisfaction and IOL exchange [3, 4]. Photic phenomena are perceived while looking at street lights or headlights of automobiles at night. Glare is defined as reduced sharpness of vision under bright light or a dim disk of light. A halo is defined as a blurred circle surrounding the image of the light source. Starburst is defined as rays radiating outward from the central point of the source of light [3–8].

The occurrence of photic phenomena in eyes with presbyopia-correcting IOLs varies greatly for each IOL type, with approximately 6.3%–75% in bifocal IOLs [9, 10], 10%–40% in extended depth of focus (EDOF) IOLs [11, 12], and 20%–70% in trifocal IOLs [5, 13]. This variation may be attributed to differences in study methods. One method includes the use of questionnaires [5, 9–15], whereas another method presents a simulated image to patients and asks them to select the photic phenomena that they perceived in daily life to evaluate the various types of photic phenomena [6]. A third method uses a halometer, exposing the patients to light sources and evaluating the actual photic phenomena [7]. The two former study methods have some issues with reproducibility and reliability because they depend on the patient’s memory. Although the halometer-based method is more objective in exposing the patients’ eyes to actual light sources and evaluating their perception of photic phenomena, it cannot classify them into glare, halo, or starburst, according to the various types of presbyopia-correcting IOLs used. Thus, none of the previous study methods could be used to objectively evaluate photic phenomena and classify them into various types. In this study, we aimed to investigate the feasibility of the photic phenomena test (PPT) for the quantification of glare, halo, and starburst using our newly developed simulator, the soft Vision Simulator, which exposes patients to light sources, similar to the halometer, and reproduces photic phenomena in real time.

## Materials and methods

This prospective study covered patients with multifocal or monofocal IOLs who consulted ophthalmologists at Kanazawa Medical University Hospital and underwent the PPT between 1 month and 3 months after cataract surgery from August 2019 to April 2020. We determined the sample size required for statistical power of 80% in this study using the statistical software, Easy R version 1.37 (R Foundation for Statistical Computing, Vienna, Austria). Patients with corrected monocular visual acuity of less than 0 logMAR or posterior capsule opacification were excluded. In addition, we excluded patients who did not understand how to perform the PPT. The mean values of the photic phenomena of each group were evaluated. The PPT was conducted on one eye under distance correction with a +0.5-D lens. A white light-emitting diode with luminance of 45,241 cd/m^2^ was introduced as a light source. All patients were instructed adequately regarding the study procedure and signed an informed consent form prior to study inclusion. The study adhered to the tenets of the Declaration of Helsinki and was approved by the Ethics Review Committee of Kanazawa Medical University (approval no. I278).

### Vision Simulator

**Fig 1:**
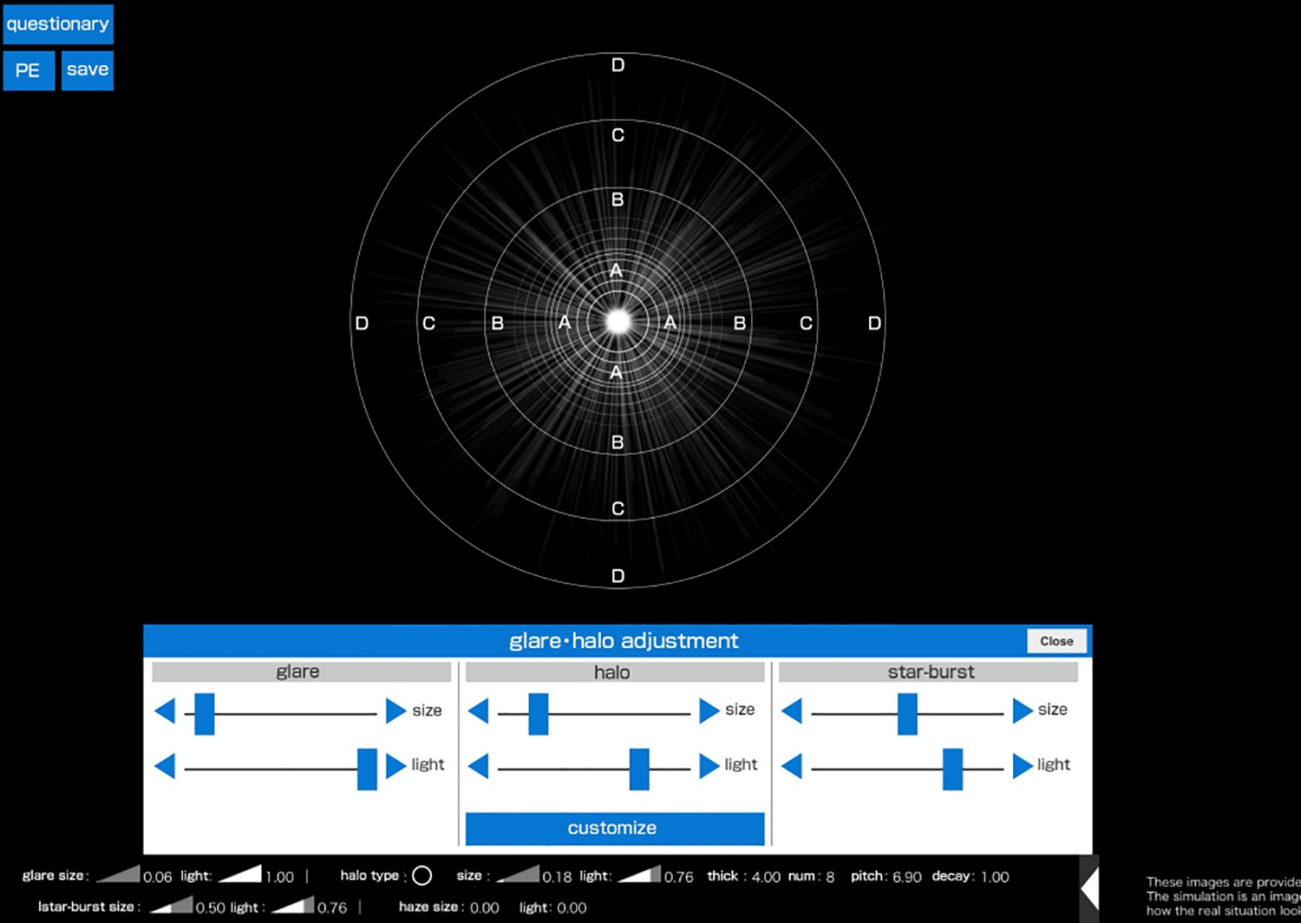
Vision Simulator screen for glare–halo adjustment. This simulator could reproduce the size and intensity of the glare, halo, and starburst by moving the slide bars and changing the images. Each reproduced image is converted into numeric values and displayed at the bottom of the screen.

The PPT was conducted using the Vision Simulator, which enables the reproduction of the patient’s perception of the light source (Fig 1). Three subjective perceptions of photic phenomena (i.e., glare, halo, and starbursts) were reproduced around the light sources. The Vision Simulator could adjust the size and intensity of glare and starbursts. Moreover, it could adjust the size, intensity, number, ring width, interval, and shape of the halo. The halo size is defined as the diameter of the whole halo. The halo ring width is defined as the breadth of each halo ring. There are sliders on the Vision Simulator screen with which one can zoom in or out. The Vision Simulator can display images on tablet and computer screens with an Internet connection. We conducted the PPT with the Vision Simulator on a 12.9-inch tablet screen.

The patient’s perception of photic phenomena could be reproduced by moving the slide bars from side to side during the PPT (Fig 1). The Vision Simulator can adjust the size of the glare and halo as well as the length of the radiating ray of the starburst. It can visualize the patient’s perception of intensity by changing the light intensity for the three types of photic phenomena (Fig 1). The size and intensity are converted into numeric values ranging from 0.01 (minimum) to 1.00 (maximum) on a visual scale of 1 to 100 at regular intervals (1.00 is equivalent to a hundred times 0.01).

**Fig 2:**
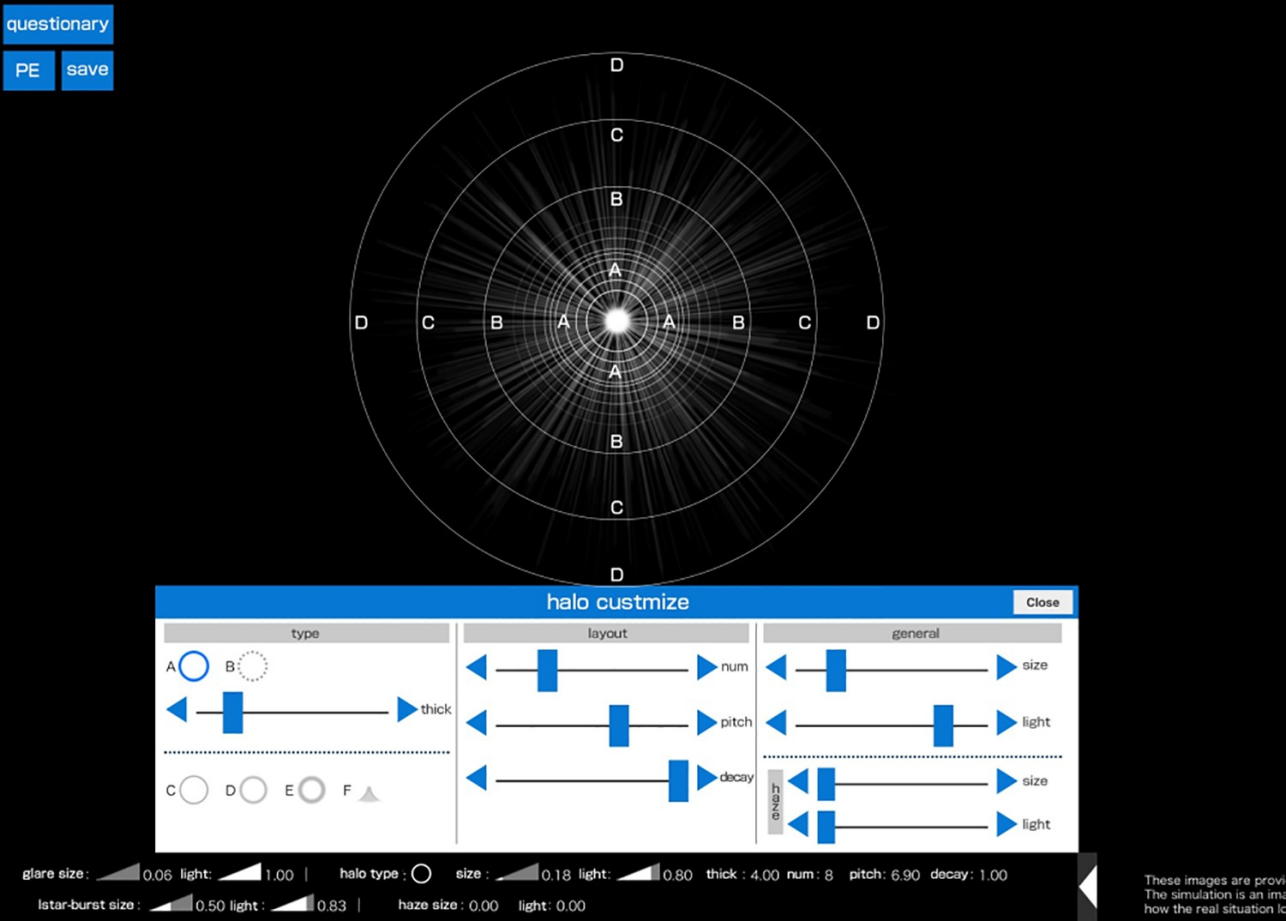
Vision Simulator screen for detailed settings for the halo. This simulator enables detailed settings for the halo; the number of rings, ring width, and interval between the rings can be reproduced.

In addition to the size and intensity, the number, ring width, and interval of the halo can be adjusted on the Vision Simulator by pressing a customized button displayed below the slide bars (Fig 2).

The number of halo rings can be adjusted from 1 to 30, and the width of each ring can be adjusted from 1.00 to 20.00. If the first halo ring is fixed, the second and the subsequent halo rings can be adjusted at intervals ranging from 1.00 to 10.00.

### PPT with the Vision Simulator

**Fig 3:**
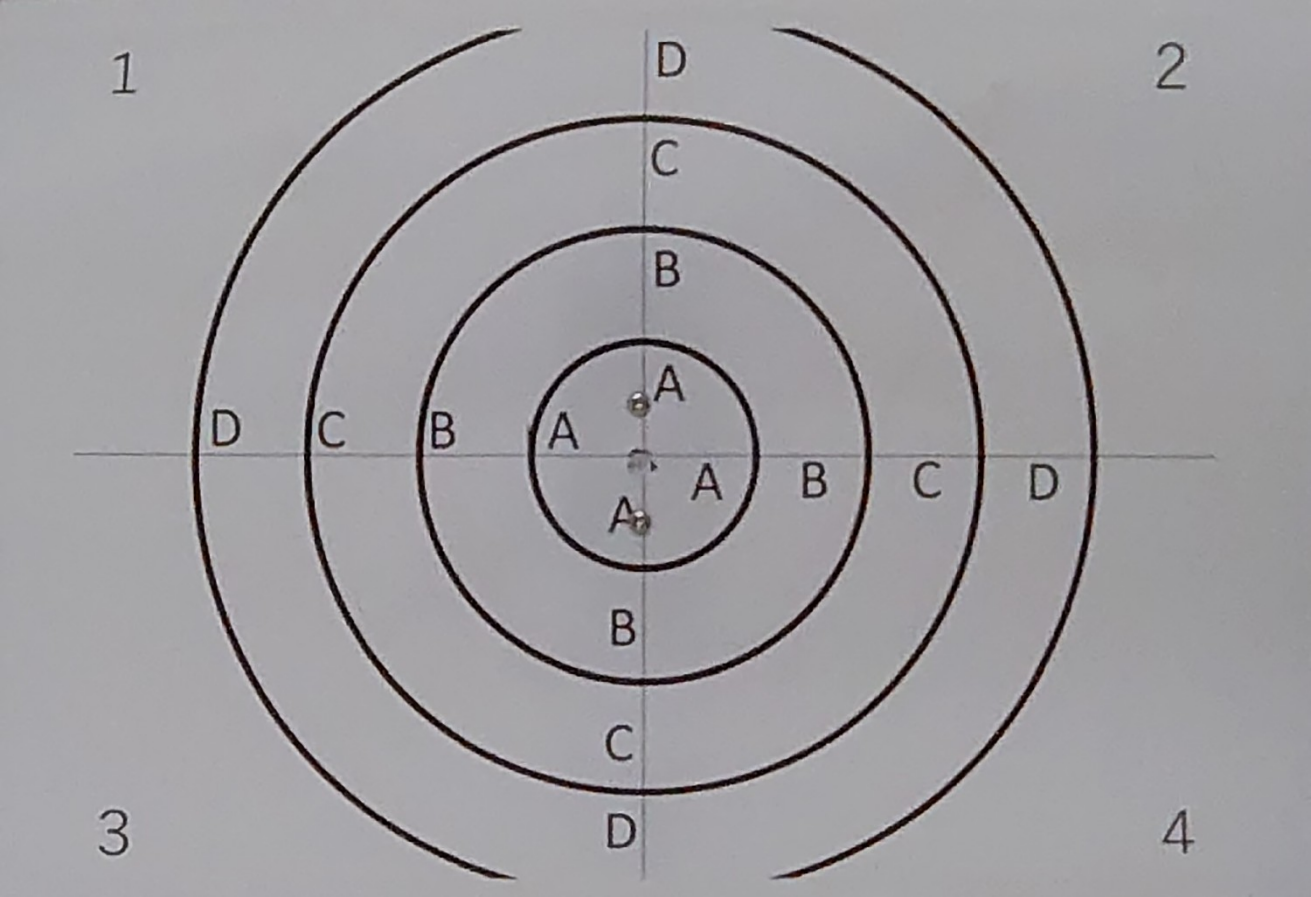
Gradations projected onto a Vision Simulator screen. These gradations were marked around the light sources at intervals of 5 cm from the center of the halo ring.

A white light-emitting diode placed 2 m away from the patient’s eye and 30 cm under his/her eye line provided the light source for the Vision Simulator during the PPT. The light in the examination room was adjusted to 7 lux at the sunset illumination level. Photic phenomena were measured by placing a +0.5-D spherical lens on the patient’s eye, corresponding to a 2-m examination distance, while the contralateral eye was shielded. Gradations were marked around the light sources at intervals of 5 cm from the center of the halo ring. These gradations were projected onto a Vision Simulator screen, as the standard for the quantitative evaluation of the size of each photic phenomenon (Figs 1–3). The patient’s actual perception of photic phenomena was reproduced in real time, as the examination was conducted by comparing light sources and the Vision Simulator screen simultaneously. The size and intensity of the reproduced images were converted into numeric values and displayed at the bottom of the screen (Fig 1). The light intensity could be displayed on a scale of 1–7 (i.e., 11,710–77,663 cd/m^2^, respectively).

The procedure of the PPT was as follows. First, we asked the patients to confirm the position of the light sources and graduators around them before making the examination room mesopic. We subsequently presented the preset images of all photic phenomena on the Vision Simulator as default at the beginning of the examination. Finally, we adjusted and reproduced the patient’s perception of photic phenomena by comparing these images and the actual light sources.

First, the size and intensity of the glare were determined while reproducing the photic phenomena. At this time, the size was determined by referring to the gradations around the light sources and Vision Simulator. The number of rings comprising the halo was determined, followed by the reproduction of the size, interval between rings, ring width, and intensity. Second, the size and intensity of the starburst were determined. The Vision Simulator enabled accurate evaluation of patients’ perception of photic phenomena, as the size of the halo and starbursts was determined with reference to the gradations. Finally, the examination was conducted after determining all the parameters and recording their values. We used the reproduced images and gradations instead of their default counterparts to accurately determine the differences between the first and second conditions for subsequent examinations under other conditions. The examiner reverted the photic phenomena to default on the screen at the beginning of examination of the contralateral eye. The PPT may enable the assessment of the gradual change in photic phenomena and preoperative prediction of their occurrence by analyzing the correlation between the optic parameters of the eyeball and postoperative photic phenomena.

### Assessment of reproducibility

We assessed the reproducibility and reliability of the PPT among patients and examiners. This assessment included 39 multifocal IOL-implanted eyes of 20 patients (mean age: 62.1 ± 8.4 years) who consulted ophthalmologists at Kanazawa Medical University Hospital and underwent the PPT from December 2019 to April 2020. Patients with ocular diseases, with the exception of refractive errors, were excluded. We conducted a reproducibility and reliability assessment for patients who were measured by one examiner twice on the same day.

Two examiners measured the same patient on the same day to assess the inter-rater reproducibility and reliability. The PPT was conducted under correction of +0.5-D hyperopia with a 45,241-cd/m^2^ light source. The following parameters were evaluated: size of glare; size, intensity, and ring width of the halo; size and intensity of the starburst.

Bland–Altman plots were used for the analysis of reproducibility. First, Bland–Altman plots were generated by plotting the differences between two measured values on the Y-axis versus the mean of two measured values on the X-axis. Subsequently, the 95% confidence intervals (CIs) of the average differences in the two measured values were calculated to determine the presence of additional errors. In cases where the interval did not include 0, a fixed bias was judged to exist with a unidirectional distribution of the measured values. Furthermore, the regression formula was calculated from the Bland–Altman plots to confirm the presence of proportional bias, and the significance of regression was estimated. A proportional bias was judged to exist when the regression was judged to be significant. The intraclass correlation coefficients (ICCs) were used to examine the inter-rater and inter-patient reliability between the measured PPT values. The statistical software Easy R version 1.37 (R Foundation for Statistical Computing, Vienna, Austria) was used to determine the 95% CIs and significance of regression.

### Comparison of photic phenomena between two presbyopia-correcting IOLs and a monofocal IOL

Analysis of variance with Tukey’s test was used to estimate the presence of statistically significant differences in average ages and each parameter (i.e., size of glare; size, intensity, and ring width of the halo; and size and intensity of the starburst) among the three lenses. P-values <0.05 were considered statistically significant.

## Results and discussion

This study included 111 multifocal and monofocal IOL-implanted eyes of 111 patients (mean age: 65.1 ± 9.0 years) who underwent the PPT 1 month postoperatively. Of these, 30 eyes of 30 patients (mean age, 63.8 ± 11.0 years) were implanted with EDOF IOLs (TECNIS Symfony; Johnson & Johnson Vision, Santa Ana, CA, USA), 50 eyes of 50 patients (mean age, 65.0 ± 8.1 years) were implanted with trifocal IOLs (AcrySof IQ PanOptix Trifocal; Alcon, Geneva, Switzerland), and 31 eyes of 31 patients (mean age, 66.5 ± 6.9 years) were implanted with monofocal IOLs (Clareon AutonoMe; Alcon, Geneva, Switzerland).

### Reproducibility and reliability evaluation

Table 1 summarizes the presence of fixed and proportional biases in the results of the PPT for patients and examiners. Bland–Altman plots revealed the absence of both fixed bias at each point and significance of each regression line.

**Table 1:** Fixed and proportional biases in the results of the PPT.

Parameter		Fixed bias		Proportional bias	
		95% CI	Presence of bias	Inclination of the regression line	Presence of bias
Glare size	Inter-patient	−0.21 to 0.23	None	0.01	None
	Inter-rater	−0.12 to 0.21	None	−0.03	None
Halo size	Inter-patient	−0.08 to 0.22	None	0.07	None
	Inter-rater	−0.24 to 0.36	None	0.04	None
Halo intensity	Inter-patient	−0.22 to 0.39	None	0.08	None
	Inter-rater	−0.15 to 0.28	None	0.07	None
Halo ring width	Inter-patient	−0.07 to 0.11	None	0.02	None
	Inter-rater	−0.06 to 0.25	None	0.10	None
Starburst size	Inter-patient	−0.36 to 0.13	None	−0.11	None
	Inter-rater	−0.30 to 0.06	None	−0.13	None
Starburst intensity	Inter-patient	−0.38 to 0.19	None	−0.09	None
	Inter-rater	−0.14 to 0.43	None	0.14	None

Table 1 summarizes the presence of fixed and proportional biases in the results of the PPT for patients and examiners using Bland–Altman plots.

CI = 95% confidence interval.

Table 2 summarizes the ICCs between the inter-rater and inter-patient reliability and the measured PPT values. Each parameter showed high reliability.

**Table 2:** ICCs between the inter-rater and inter-patient reliability and the measured PPT values.

Parameter		ICC
Glare size	Inter-patient	0.91
	Inter-rater	0.93
Halo size	Inter-patient	0.96
	Inter-rater	0.81
Halo intensity	Inter-patient	0.84
	Inter-rater	0.90
Halo ring width	Inter-patient	0.98
	Inter-rater	0.94
Starburst size	Inter-patient	0.87
	Inter-rater	0.91
Starburst intensity	Inter-patient	0.83
	Inter-rater	0.83

Table 2 summarizes the ICCs between the inter-rater and inter-patient reliability and the measured PPT values for each parameter.

oICC = intraclass correlation coefficient.

### Comparison of the photic phenomena of two presbyopia-correcting IOLs

Table 3 summarizes the mean age of the patients according to IOL type and the mean measured photic phenomena values in the PPT for each lens.

**Table 3:** Mean age of the patients according to IOL type and mean measured photic phenomena values in the PPT.

IOL type	Mean age	Number of eyes	Mean PPT values					
			Glare size	Halo size	Halo intensity	Halo ring width	Starburst size	Starburst intensity
Symfony	63.8 ± 11.0	30	0.06 ± 0.02	4.23 ± 0.98	0.45 ± 0.13	4.96 ± 2.44	0.46 ± 0.11	0.65 ± 0.15
PanOptix	65.0 ± 8.1	50	0.05 ± 0.01	1.94 ± 0.66	0.60 ± 0.22	6.58 ± 3.72	0.41 ± 0.12	0.57 ± 0.18
Clareon	66.5 ± 6.9	31	0.06 ± 0.02	0.00	0.00	0.00	0.42 ± 0.19	0.69 ± 0.27

IOL = intraocular lens.

**Fig 4:**
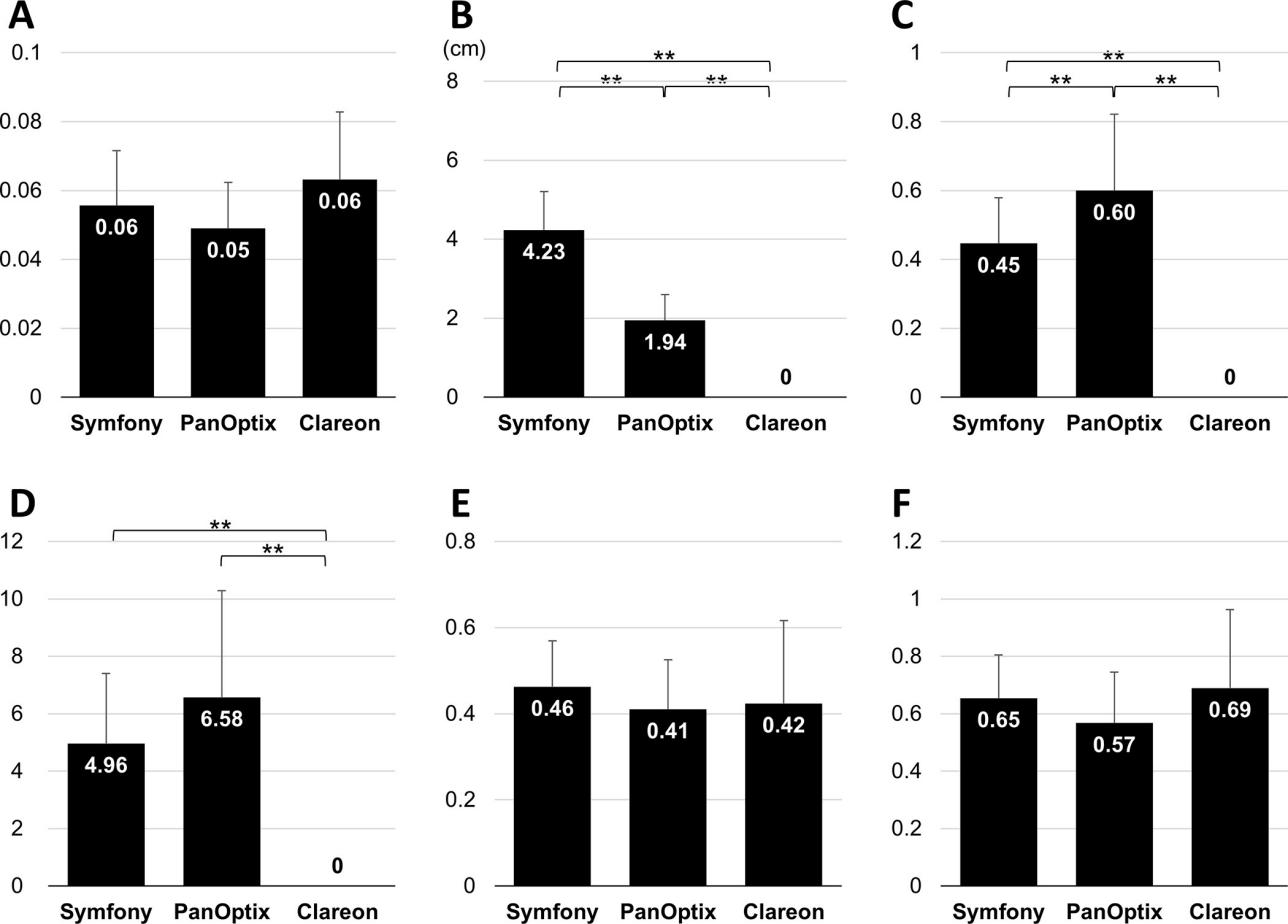
Mean value of the (A) glare size, (B) halo size, (C) halo intensity, (D) halo ring width, (E) starburst size, and (F) starburst intensity for each IOL group.

**Fig 5:**
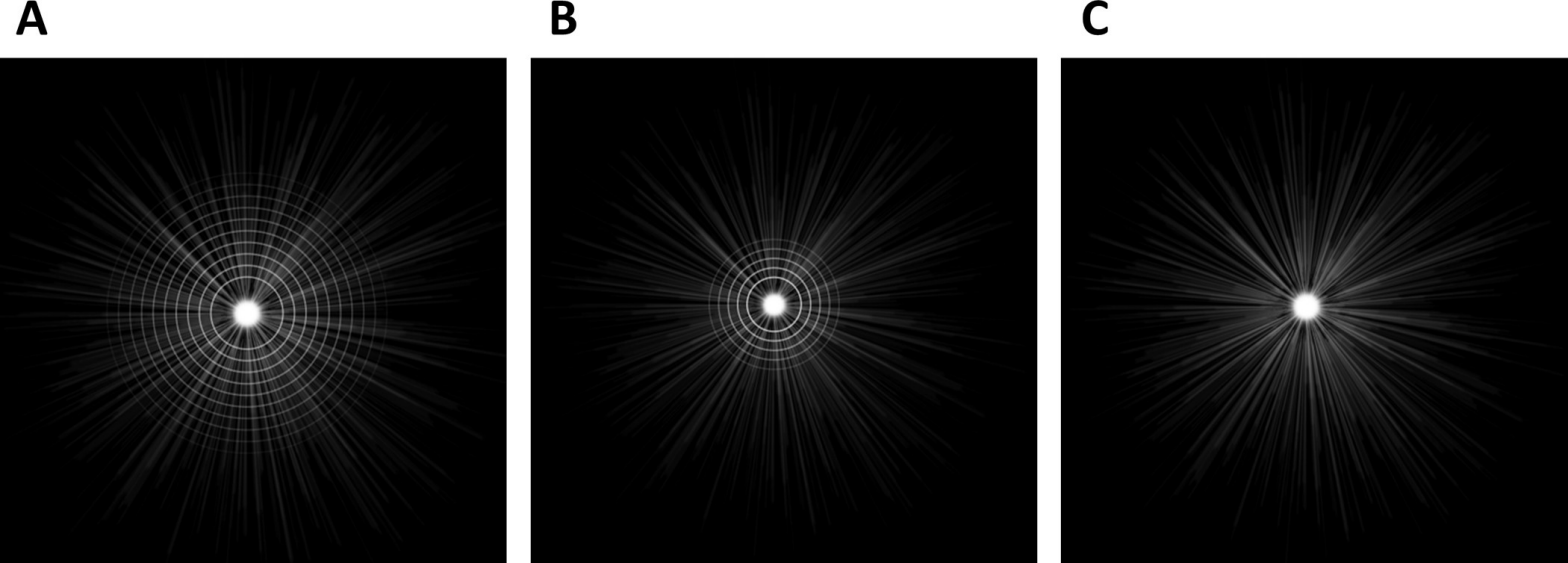
Mean images of the photic phenomena of the (A) Symfony, (B) PanOptix, and (C) Clareon groups on the Vision Simulator. These are based on the mean values of various parameters.

There were no significant differences between the ages of the groups. The mean values of each photic phenomenon parameter in the PPT are summarized in Figs 4 and 5. LV4 indicated a luminance level of 45,241 cd/m^2^. There were no significant differences in the size of the glare. The halo was significantly larger in the Symfony group than in the PanOptix group (p < 0.01), but the halo was significantly brighter in the PanOptix group (p < 0.01) than in the Symfony group. In contrast, no halos were perceived in the Clareon group. The starburst size and intensity did not differ significantly between the Symfony, PanOptix, and Clareon groups.

The mean images of various photic phenomena of eyes with IOL implantation on the Vision Simulator, based on the mean values of various parameters, are summarized in Fig 5A–5C. A salient observation was that eyes with Symfony IOLs perceived glares, starbursts, and halos simultaneously, whereas those with PanOptix IOLs perceived clear halos around the glares and starbursts simultaneously. Furthermore, those with Clareon IOLs perceived glares and starbursts simultaneously, but no halos.

We confirmed that the PPT could assess the three different types of photic phenomena, namely, glare, halo, and starburst. Furthermore, the PPT could identify each photic phenomenon characteristic of EDOF, trifocal, and monofocal IOLs. We found that the PPT was highly effective for the quantitative and subjective assessment of photic phenomena of various IOLs.

Photic phenomena, such as glare, halo, and starbursts, are common visual complaints in patients with multifocal IOLs, especially while looking at headlights of automobiles and street lamps when driving at night. This has a detrimental effect on the patients’ postoperative satisfaction [3–5, 10, 16].

Various studies, which were mostly conducted using questionnaires, have reported photic phenomena, with inconsistent results [9–15, 17]. Tan et al. reported no significant differences between the evaluation results of photic phenomena questionnaires in participants with Symfony IOLs implanted in both eyes in the micro-monovision and binocular emmetropia groups [11]. In contrast, Cochener reported that patients with micro-monovision implanted with the Symfony IOL exhibited more severe photic phenomena than those with binocular emmetropia [12]. Ribeiro and Ferreira reported that the percentage of photic phenomena perception was 20% in patients with PanOptix IOL implants [5], but Bissen-Miyajima et al. reported a higher halo perception rate of 70% [13]. We assumed that the results of questionnaire-based studies were highly dependent on the patients’ memory of their perception of photic phenomena and the frequency of going out at night. The PPT may enable the evaluation of the characteristics, degree, and gradual change of photic phenomena in various IOL types with greater reliability. Furthermore, the PPT evaluation on the correlation between the patient’s preoperative ocular parameters, such as corneal shape, higher-order aberrations, and pupil width, and the measured PPT results may contribute to the future identification of risk factors for postoperative photic phenomena.

Few studies have investigated photic phenomena using simulators and electronic media. Son et al. reported an investigation method using a halo and glare simulator [6]. This instrument could adjust and project the size and intensity of the glare and halo and adjust the type of starburst (e.g., starburst integrated with halos). However, we found it difficult to assess the photic phenomena of various IOL types in detail with that instrument, as it did not allow for continuous adjustment of the ring width or interval of the halo, nor could the size and intensity of the starburst be adjusted independently. Furthermore, this assessment method depended on the patient’s memory without having them look at actual light sources. Therefore, its reliability and reproducibility were limited, as the current study sample included middle-aged and older patients with presbyopia-correcting IOLs.

Buckhurst et al. reported the halometer method, which evaluated photic phenomena by presenting actual light sources to the participants, similar to the PPT [7]. This instrument displayed letters on a tablet screen with light sources at the center of the screen and specified the extent of the photic phenomena by the number of decipherable letters. Interestingly, the PPT was more effective in evaluating the respective size, intensity, and ring width of the halo, glare, and starburst independently.

In this study, the PPT was conducted on eyes implanted with monofocal IOLs as a control group. The results showed that there were no significant differences in the perception of the glare between the two presbyopia-correcting IOL groups and the monofocal IOL group and that there was no perception of halo in the monofocal IOL group. Song et al. reported that some eyes with monofocal IOLs perceived a slight halo in their questionnaire study [18]. These perceptions of halo may be attributed to the influence of myopia and astigmatism. The influence of refractive error and astigmatism were examined in this study, as the PPT was conducted under a refractive correction environment.

However, this study had several limitations. First, the examination distance (2 m) was lower than that between the patient and the light sources in daily life. The PPT was conducted in an examination room at a distance of 2 m, while photic phenomena are usually perceived when looking at faraway objects, such as street lights or car headlights. It should be noted that examination distances that are closer to the light source may influence and change the perception of photic phenomena, which may be inconsistent with the phenomena perceived in daily life. We also conducted a preliminary study to compare the results of the PPT and questionnaires on the perception of photic phenomena in daily life. We confirmed that there was a correlation between the results of these assessments. The PPT enabled a detailed assessment of photic phenomena without dependence on patients’ memory by presenting light sources and reproducing their perception on the tablet screen. Therefore, it was necessary to inform patients concerning the examination method beforehand and explain that the examination period would be prolonged, usually 10–15 min for each eye, to achieve credible results. Second, the size of the study sample was small. Further studies with a larger sample size are required to confirm and evaluate the chronological changes in and the influence of refractive error and astigmatism on each characteristic of the photic phenomena in various IOLs.

## Conclusions

Our results showed that the PPT demonstrated high reproducibility for detailed quantification of photic phenomena. This quantification enabled the identification of the characteristics of photic phenomena in patients with various presbyopia-correcting IOLs. The quantitative assessment of photic phenomena with the PPT may improve the prediction of gradual changes and the identification of related risk factors.

## Supporting information

S1 Table The mean values of the photic phenomena for each IOL group. S1 Table shows the mean values of the photic phenomena for Symfony, PanOptix and Clareon.
(PDF)
