# Supplementary material for: Quantitative assessment of photic phenomena in the presbyopia-correcting intraocular lens
Source: PLoS One. 2021 Dec 1;16(12):e0260406. doi: 10.1371/journal.pone.0260406 (PMC8635348; doi:10.1371/journal.pone.0260406)
Supplement: S1 Table — shows the mean values of the photic phenomena for Symfony, PanOptix and Clareon. (PDF) [file pone.0260406.s001.pdf]

S1 Table. The mean values of the photic phenomena for each IOL group

IOL group	Mean age (years ± SD)	Number of eyes	Mean PPT values					
			Glare	Halo			Starburst	
			Size ± SD	Size (cm) ± SD	Intensity ± SD	Ring width ± SD	Size ± SD	Intensity ± SD
**Symfony**	65.1±9.0	30	0.06±0.02	4.23±0.98	0.45±0.13	4.96±2.44	0.46±0.11	0.65±0.15
**PanOptix**	65.0±8.1	50	0.05±0.01	1.94±0.66	0.60±0.22	6.58±3.72	0.41±0.12	0.57±0.18
**Clareon**	66.5±6.9	31	0.06±0.02	0	0	0	0.42±0.19	0.69±0.27

IOL, intraocular lens; SD, standard deviation; PPT, photic phenomena test
